# Sleep architecture and cognitive changes in olanzapine-treated patients with depression: A double blind randomized placebo controlled trial

**DOI:** 10.1186/1471-244X-14-202

**Published:** 2014-07-17

**Authors:** Lauren K Lazowski, Ben Townsend, Emily R Hawken, Ruzica Jokic, Regina du Toit, Roumen Milev

**Affiliations:** 1 Centre for Neuroscience Studies, Queen’s University, Kingston, Canada; 2 Department of Psychology, Carleton University, Ottawa, Canada; 3 Department of Psychiatry, Queen’s University, 752 King Street West, Kingston, ON K7L 4X3, Canada

**Keywords:** Olanzapine, Sleep continuity, Slow-Wave sleep, Depression, Cognitive function, Illness severity

## Abstract

**Background:**

Disturbance in sleep quality is a symptom of Major Depressive Disorder (MDD) and Bipolar Disorder (BD) and thus improving quality of sleep is an important aspect of successful treatment. Here, a prospective, double-blind, randomized, placebo-controlled study examined the effect of olanzapine (an atypical antipsychotic) augmentation therapy on sleep architecture, specifically slow wave sleep (SWS), in the treatment of depression. The effect of olanzapine augmentation therapy on other features of sleep (e.g., sleep continuity) and depression (e.g., illness severity and cognitive function) were also determined.

**Methods:**

Patients currently experiencing a major depressive episode and who were on a stable medication were included. Sleep architecture was measured by overnight ambulatory polysomnography. Illness severity was determined using the Montgomery-Asberg Depression Rating Scale (MADRS). Cognitive function was examined using Cambridge Neuropsychological Test Automated Battery (CANTAB): Spatial Working Memory (SWM), Spatial Span (SSP), and Reaction Time (RTI) tasks. Polysomnographs, clinical measures and cognitive tests were administered at baseline, after 2–4 days of treatment and after 28–31 days of treatment. Twenty-five patients participated in the study (N = 10, N = 15 for placebo and olanzapine treated groups respectively).

**Results:**

The primary objective of the study was to assess the objective (polysomnographic) changes in sleep quality, defined as changes in SWS, following olanzapine treatment for depression. Latency to but not duration of SWS was found to significantly differ between olanzapine- and placebo-treated participants (Hedge’s g: 0.97, 0.13 respectively). A significant improvement in olanzapine-treated participants over placebo-treated participants was observed in secondary outcome measures, including sleep efficiency, total sleep time, and sleep latency. Secondary objectives assessed the subjective changes in sleep quality parameters and correlated them with measures of illness severity and changes in cognition. MADRS scores were significantly improved in olanzapine-treated participants over time but not more than placebo treatment. There was no significant difference between olanzapine- and placebo-treated participants in SWM, SSP or RTI tasks.

**Conclusions:**

Olanzapine augmentation treatment generally did not improve SWS but did improve sleep continuity and depression. Olanzapine may be one of few medications that improve sleep continuity, thus directly targeting symptoms of depression.

**Trial registration:**

ClinicalTrials.gov, NCT00520507.

## Background

Disturbance in sleep quality is a symptom of Major Depressive Disorder (MDD) and Bipolar Disorder (BD) [1], with MDD having the strongest association with sleep disturbances [2,3]. It is well documented that the severity of the sleep disturbance is related to treatment response, as well as the chronicity of the disorders [4,5], and as such, is an important aspect of treatment.

Sleep has two main components: Rapid Eye Movement (R) and Non-REM (N) sleep [6]. N sleep includes stages 1 to 3, where stage 3 is referred to as slow wave sleep (SWS). SWS is considered the deepest stage of sleep and involves a higher arousal threshold [7]. This is the most prevalent stage of the sleep cycle in the first third of the night, approximately 10%-15% of total sleep time (TST). As SWS shortens in each cycle, the amount of R sleep increases, with R sleep being most prevalent during each cycle in the final third of the night, constituting 20%-25% of TST [6].

Sleep architecture in patients experiencing depression is generally thought to be altered [8]. Sleep during depressive episodes is characterized by a reduction in SWS, or increased latency to SWS, increased duration of R sleep, shortened latency to R sleep, continuity disturbances, and decreased TST [9-12]. Altered SWS and R sleep are seen in approximately 50% of patients with affective disorders [12]. Sharpley *et al.*[13], found a negative association between SWS activity and illness severity of depression; however, variables such as sleep efficiency and TST were also found to significantly increase as illness severity decreased.

Cognitive deficits in MDD and BD include working memory, attention and psychomotor speed [14-18]. Acute and chronic sleep deprivation has been shown to affect attention, working memory and cognitive function [19-22]. In healthy volunteers, SWS deprivation was associated with impaired cognitive performance and a delayed reaction time upon awakening [23,24]. In particular, studies have shown that duration of SWS per night is correlated with working memory performance in elderly and schizophrenic samples [25,26]. However, the role of sleep in the cognitive performance of patients with mood disorders is unknown.

Monoaminergic neurotransmitters are thought to be involved in the relationship between sleep and depression. The serotonergic [27,28], cholinergic [6], histaminergic [29,30], and noradrenergic [27] systems all contribute to the pathophysiology of depression and the underlying mechanisms of sleep. Antidepressants can produce changes in the sleep electroencephalogram (EEG) of depressed patients, the most prominent being the suppression of R sleep. This is thought to occur by increasing norepinephrine (NA) and/or serotonin (5-HT) function [31]. 5-HT receptors have also been shown to play a critical role in the regulation of SWS. Several antidepressants, particularly tricyclic antidepressants and trazodone, are thought to normalize SWS because of their affinity for 5-HT_2A_ and 5-HT_2C_ receptors [31].

It has been estimated that up to 30% of patients with depression fail to respond to traditional antidepressant treatments alone [32]. Augmentation of antidepressant therapy, with olanzapine, an atypical antipsychotic, is often used in routine clinical practice for the treatment of depression. Olanzapine has affinity for dopamine D_1_, D_3_, and D_4_; serotonin 5-HT_2A/C_, 5-HT_3_ and 5-HT-_6_; muscarinic M_1_-M_5_; adrenergic α_1_; and histamine H_1_ receptors. Moreover, olanzapine is an antagonist at these sites, with highest affinity for both the dopamine and serotonin receptors [33].

To date, many studies have focused on improving illness severity of patients with MDD or BD by combining mood stabilizing medication with a change in sleep-related behaviours [34-36]. A review by Riemann *et al*. [8] described several different interventions used to improve sleep habits of patients with MDD, such as psychotherapy [37]*,* pharmacological treatment [38], and sleep deprivation therapy [39], among others. A large majority of these studies, regardless of treatment type, found that improving sleep continuity measures resulted in improvements in mood. Olanzapine has been shown to increase sleep continuity, subjective sleep measures and SWS in healthy volunteers [40-42] while an association between changes in sleep architecture and mood improvement in patients with MDD remains unclear [13]. Finally, Olanzapine has also been shown to improve cognition in schizophrenic patients [43]; however, its impact on cognition in depressive patients has not yet been fully described.

The primary aim of this study was to examine the effects of olanzapine augmentation treatment on sleep architecture, specifically SWS, in patients experiencing a major depressive episode. Furthermore, we examined several secondary outcome measures to determine the effects of olanzapine augmentation on other elements of sleep, like TST and sleep continuity, and aspects of depression, including illness severity and cognitive function.

## Methods

The present study was a prospective, double-blind, placebo-controlled, repeated measures, polysomnographic study. This study was approved by the Queen’s University research ethics board, Health Canada, and was registered with clinicaltrials.gov (NCT00520507).

### Participants

A 15% change in the primary outcome measure, SWS, was selected to be a clinically significant and meaningful change; this effect size was used to calculate sample size, indicating that 30 participants (15 placebo, 15 treatment) were needed to have enough power to detect an effect of olanzepine on SWS. To account for drop-outs, we aimed to enroll 40 participants. The trail was stopped, however, before reaching recruitment goals (May 2009) due to an inability to recruit participants. Only thirty-one participants signed informed consent to participate in this study.

Participants were screened and enrolled into the study (beginning October 2007) by blinded clinicians (nurses) and recruited from tertiary care mood disorders units, general practitioners offices, and from the community. All were 18 years of age or older, and met DSM-IV-TR criteria [44] for MDD, Bipolar I Disorder, Bipolar II Disorder or Bipolar Disorder NOS, as confirmed by the Mini International Neuropsychiatric Inventory (MINI) [45]. At enrollment, participants were experiencing a major depressive episode (MDE), defined as a Hamilton Depression Rating Scale-17 item (HDRS-17) score of >15, and not a mixed episode, defined as a Young Mania Rating Scale Score (YMRS) of ≤ 12 [46]. Excluded criteria were current or past diagnosis of schizophrenia or dementia, substance abuse within three months of enrolment (excluding caffeine or nicotine), imminent risk of suicide or danger to themselves or others, known intolerance for olanzapine, a serious or inadequately treated medical illness, a history of seizures, previous enrollment in the study or enrollment in another treatment study within 4 weeks prior. Participants could not be taking any other antipsychotic medication at the time of enrolment and must have been on a stable dose of all medications for 4 weeks prior to enrolment. Benzodiazepines and other sleep aids were discontinued if a stable dose had not been achieved for four weeks prior to enrolment (number of participants on each concomitant drug: Placebo: 1 Remeron, 2 Prozac, 1 Valproic Acid, 2 Clonazepam, 1 Lorazepam, 1 Trazodone, 1 Citalopram, 1 bupropion, 1 fluvoxamine, 1, cipralex, 1 oxycontin, 1 Ativan, 1 Topamax, 1 fluvianzine; Olanzepine: 2 Remeron, 2 Prozac, 1 doxepine, 1 amitriptyline,1 oxazepam, 5 Clonazepam, 1 ritalin, 1 Lorazepam, 1 lithium, 1 immovane 1 Citalopram, 2 bupropion, 1 fluvoxamine, 2 cipralex, 2 Effexor). Four participants failed baseline screening (1 had a HAMD score that was too low, 1 was already receiving olanzepine treatment [and also had a too-low HAMD score], and 2 failed to show at the screening appointment) and two participants withdrew (refused to continue to participate) between randomization and day 2–4, and were not included in the analysis. Thus, 25 participants were included in the analysis. Three participants from the olanzapine-treated group terminated from the study before day 28–31, due to a worsening of original mood symptoms. Participants were monitored for sleep apnea and/or hypopnea throughout the course of the study as the presence of these conditions at any point warranted exclusion from the study due to the confounding nature of these events on sleep architecture. No participants needed to be removed due to obstructive sleep apnea/hypopnea.

### Clinical measures

Participants were assessed at three time-points by a clinician blinded to treatment group: baseline (before randomization and administration of study medication), 2–4 days, and 28–31 days after the administration of study medication. Each clinical assessment consisted of the HDRS-17 [47], the Montgomery Asberg Depression Rating Scale (MADRS) [48], the Hamilton Anxiety Rating Scale (HARS) [49,50], the YMRS [46], the Pittsburgh Sleep Quality Index (PSQI) [51], Visual Analogue Scale (VAS) for sleep quality [52], and the Epworth Sleepiness Scale (ESS) [53]. At baseline, the MINI and the Clinical Global Impression-Severity (CGI-S) [54], scales were administered. During day 28–31, the Clinical Global Impression-Improvement (CGI-I) was administered. Baseline blood work, physical exam, and pregnancy test (in women of childbearing potential) were performed.

### Neurocognitive measures

At each visit, participants completed cognitive testing using three Cambridge Neuropsychological Test Automated Battery (CANTAB) tasks, including: Spatial Span (SSP), Spatial Working Memory (SWM) and Reaction Time (RTI) [55,56] (Cambridge Cognition, Cambridge, UK; http://www.camcog.com). One participant did not complete the CANTAB testing.

### Medication

Participants were randomly assigned to either placebo or olanzapine (orally disintegrating, zydis, formulation) conditions. A randomization table was generated by a statistician to randomize for order that then placed sealed envelopes containing a single allocation (A:Placebo, B:Olanzapine) into a box in the order specified by the randomization table. Following screening, the participant was instructed to pull the next envelope in the box. This allocation was given to an unblinded pharmacist who filled a prescription that read ‘placebo/olanzapine.’ Medication dosing started at 2.5 mg on day 1 and increased to 5 mg at day 2; during day 2–4, the dosing was titrated up or down, in increments of 2.5 mg to a maximum of 20 mg. The mean dose of olanzapine was 6.67 mg at the end of the study; doses ranged from 5 mg to 10 mg. Eight participants in the placebo-treated group and twelve in the olanzapine-treated group were taking at least one antidepressant as concomitant medications; concomitant antidepressants included amitriptyline, bupropion, escitalopram, citalopram, doxepine, venlafaxine, fluoxetine, remeron, and trazodone. One participant in the placebo-treated group was taking only a benzodiazepine; one participant in the ola\nzapine-treated group was only taking one mood stabilizer; one participant in the placebo-treated group and two in the olanzapine-treated group were not taking any other psychotropic medications. All concomitant medications were stable throughout the duration of the study.

### Polysomnographic recordings

At each time-point, an overnight sleep polysomnograph (PSG) was performed by a clinician blinded to treatment group at the participants’ home, using the MediPalm Personal Recording Device (Braebon Medical Corporation, Kanata, Ontario, Canada). The overnight sleep PSG included four electroencephalogram channels (C4-A1, C3-A2, O2-A1, O1-A2 [57]), an electro-oculogram (two channels), a submental electromyogram (EMG), a finger pulse oximetry, an oronasal airflow (oronasalthermistor), a chest and abdominal movement belt (respiratory inductance plethysmography), a vibration snore sensor and an anterior tibialis EMG. A position sensor was used to monitor position continuously (Ultima Body Position Sensor; Braebon Medical Corporation, Carp, Canada). Participants were not monitored in person. Sharpley *et al.*[31,58,59] have demonstrated that the use of home sleep recordings provides a reliable means of detecting the effects of medications on sleep architecture. Once the PSG was applied (approximately 1900 hrs each study night), a timer was set to record for eight hours following usual sleep time or until the participant rose in the morning. Usual sleep time was verbally confirmed for each participant. Participants were asked to retire and rise at their usual time and were to refrain from alcohol on study nights; however, normal caffeine and nicotine intake was maintained. While participants did not all go to bed at the same time, eight hours or less was sufficient sleep time for those enrolled. Clinicians returned in the morning (following the participants’ usual wake time) to retrieve the PSG equipment. Sleep latencies were calculated from lights out. PSGs were manually scored in 30-second epochs according to standardized criteria of American Academy of Sleep Medicine [60], using Pursuit Advanced Sleep System software (Braebon Medical Corporation, Carp, Canada). Ruehland *et al*. [61] have demonstrated that measures of sleep architecture are not affected by the number of EEG electrode placements used in scoring the PSG. Technicians were blinded to treatment status. Only one PSG recordings was excluded from analysis due to technical problems. Obstructive apneas and hypopneas, defined as partial airway obstructions leading to a 50% reduction in thoracoabdominal movement lasting for at least 10 seconds [62], were scored using the criteria from the American Academy of Sleep Medicine Task Force [63]. Arousals were scored based on American Sleep Disorders Association criteria, 1992 [57]. The respiratory disturbance index (RDI), which included apneas, hypopneas, and snore arousals for the number of events per hour of sleep, were calculated.

### Statistical analysis

PSG recording and clinical measures (with the exception of the CGI) were analyzed using 2-way and 1-way repeated measures analysis of variance (ANOVA). The design included two treatment groups (placebo × olanzapine) across three time-points (baseline, Day 2–4 and Day 28–31). Independent-samples t-tests examined post-hoc comparisons. Hedge’s g was used to calculate the effect size of the primary outcome measure, changes in SWS, between groups on Day 28–31. Missing PSG, clinical and CANTAB data were replaced with the last observation carried forward. One participant did not complete any of the CANTAB and was not included for this analysis. One-tailed distributions were used for all clinical measures, while for polysomnographic and neurocognitive measures, two-tailed distributions were used. Additionally, demographic comparisons between groups were analyzed using Fisher’s exact tests or ANOVAs (see Table 1). For repeated-measures ANOVA, trend analyses (contrasts) were performed and reported. Neither sphericity of variance nor a significant main effect of the within-subject variable is an assumption of running trend analysis.

**Table 1:** Sociodemographic characteristics

		**Placebo group (N = 10)**	**Olanzapine group (N = 15)**	
		**N**	**N**	**P-value**
**Gender (%)**				**0.69**
	Male	5 (50)	6 (40)	
	Female	5 (50)	9 (60)	
**Diagnosis (%)**				**1.0**
	MDD	5 (50)	7 (47)	
	BD	5 (50)	8 (53)	
**Age (±SD)**		46 (9)	46 (17)	**0.24**
**Weight (±SD)**	Visit 1	185 (50)	178 (36)	0.76
	Visit 4	185 (51)	182 (37)	

MDD = Major Depressive Disorder, BD = Bipolar Disorder. P-value reported for the test statistic for Fisher’s Exact Test (gender and diagnosis) and between groups ANOVA and repeated measures ANOVA (age and weight, respectively).

## Results

Twenty-five participants were included in the analysis (N = 15 olanzepine and N = 10 placebo treated). The mean (±SD) age of the olanzapine-treated group and placebo-treated group was 46 ± 17 years (range: 20–59, median: 45) and 46 ± 9 years (range: 19–79, median: 46), respectively and did not differ significantly between the treatment groups (see Table 1).

### Polysomnographic measures

**Figure 1:**
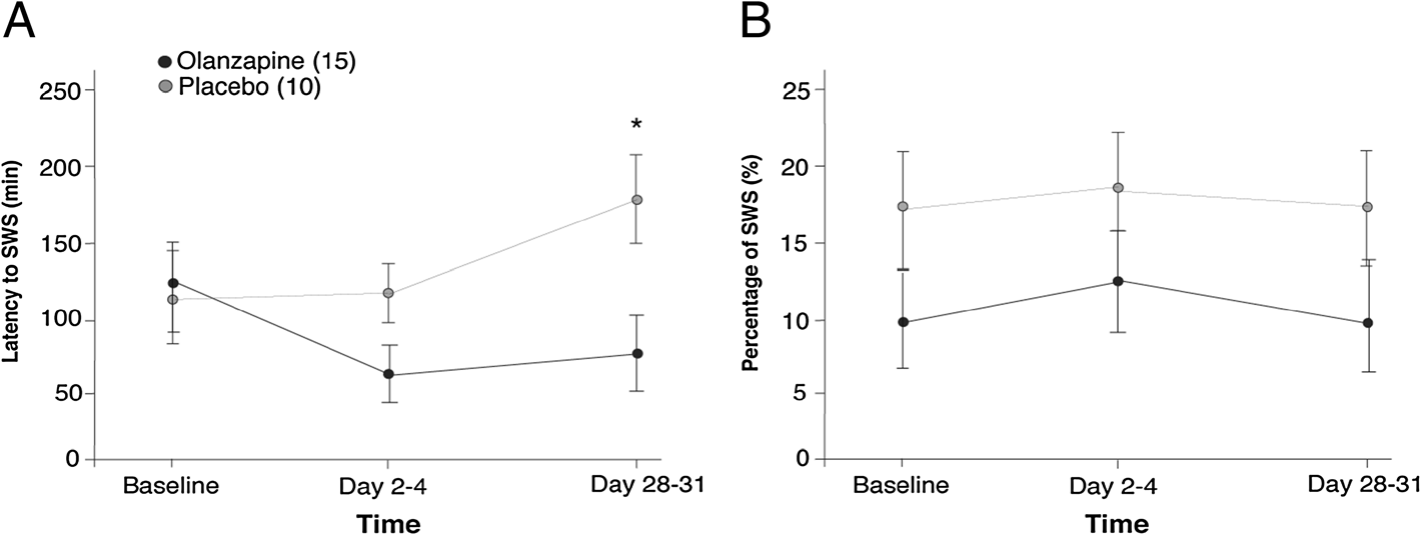
**Latency to slow wave sleep and percentage of slow wave sleep. A)** Mean ± standard error of the mean for latency to slow wave sleep (SWS) in minutes, for both olanzapine- and placebo-treated groups for each time. **B)** Percentage of SWS measured by ratio of SWS within total sleep time (TST) in minutes. *p = 0.024 between placebo and olanzapine groups on Day 28–31.

Table 2 summarizes all primary and secondary polysomnographic outcome measures for sleep architecture, in both olanzapine and placebo groups, across baseline, day 2–4, and day 28–31. Figure 1 shows the percentage of TST spent in SWS and latency to SWS in both olanzapine- and placebo-treated groups. A 2-way repeated measures ANOVA did not yield significant changes in percentage of TST spent in SWS between the olanzapine- or placebo-treated group (F[1,23] =2.36; p = 0.138; Hedge’s g = 0.53) across time (F[1,23] =0.256; p = 0.775). Latency to SWS significantly decreased with olanzapine treatment by the end of the trial (no main effect of treatment, F[1,23] = 2.49, p = .128; significant interaction of time x treatment, F[1,23] = 5.49, p = 0.028). Comparing the two treatment groups at each time point indicated that at the beginning of the trial latency to SWS was not different (t[23] = -.192, p = .849) but by Day 28–31, the olanzapine treated group reached SWS faster than the placebo group (t[23] = 2.42, p = 0.024; hedges g = 0.97). Latency to sleep however, was not significantly different and could not account for decreased latency to SWS in the olanzapine treated group (no main effect of treatment F[1,23] = 2.17, p = 0.154 or time F[1,23] = 0.297, p = .591). Duration of SWS was also not significantly affected by the olanzapine treatment compared to placebo (F[1,23] =0.581; p = 0.454) over time (F[1,23] = 0.00; p = 0.984; Hedge’s g = 0.13).

**Table 2:** Polysomnographic measures

**Sleep parameter**	**Placebo**			**Olanzapine**			
	**Baseline**	**Day 2-4**	**Day 28-31**	**Baseline**	**Day 2-4**	**Day 28-31**	**ANOVA**
	**Mean ± SD**			**Mean ± SD**			
**Sleep continuity**							
Total time in bed (min)	468 ± 27	454 ± 37	458 ± 30	460 ± 41	457 ± 32	461 ± 32	*p = .523*
Total sleep time (min)	315 ± 62	307 ± 58	289 ± 75	326 ± 168	383 ± 66	363 ± 89	*p* = .043
Total wake time (min)	153 ± 67	148 ± 63	169 ± 75	135 ± 107	74 ± 68	80 ± 63	*p* = .023
No. of awakenings	29 ± 18	23 ± 9	26 ± 11	18 ± 7.0	13 ± 4.9	15 ± 7.1	*p* = .775
No. of stage changes	145 ± 77	125 ± 50	123 ± 50	99 ± 36	101 ± 44	98 ± 38	*p* = .254
Respiratory disturbance index	15 ± 14	13 ± 12	8.7 ± 7.2	13 ± 21	11 ± 25	12 ± 25	*p* = .126
Sleep efficiency	67 ± 14	68 ± 14	63 ± 17	71 ± 23	84 ± 14	83 ± 13	*p* = .031
**Sleep architecture**							
Latency to sleep (min)	76 ± 61	86 ± 67	92 ± 78	69 ± 83	38 ± 50	41 ± 45	*p = 0.069*
Latency to Stage 1 (min)	67 ± 54	73 ± 51	85 ± 77	72 ± 74	42 ± 42	45 ± 41	*p* = .073
Duration of Stage 1 (min)	36 ± 28	30 ± 16	31 ± 16	32 ± 18	25 ± 12	27 ± 10	*p* = .984
Duration of Stage 1 (% of TST)	11 ± 8.0	10 ± 4.5	11 ± 5.0	11 ± 11	6.5 ± 2.8	7.2 ± 3.5	*p* = .353
Latency to Stage 2 (min)	82 ± 68	92 ± 72	96 ± 81	79 ± 86	46 ± 59	47 ± 49	*p* = .058
Duration of Stage 2 (min)	168 ± 62	160 ± 62	166 ± 58	199.3 ± 69.3	230.7 ± 55.4	230 ± 55	*p* = .048
Duration of Stage 2 (% of TST)	53 ± 13	51 ± 12	56 ± 12	57 ± 12	60 ± 12	60 ± 10	*p* = .927
Latency to R (min)	212 ± 88	220 ± 89	277 ± 250	165 ± 132	184 ± 100	180 ± 97	*p* = .552
Duration of R (min)	59 ± 20	66 ± 27	48 ± 28	77 ± 50	82 ± 37	87 ± 39	*p* = .285
Duration of R (% of TST)	19 ± 5.9	22 ± 7.8	16 ± 7.5	20 ± 12	21 ± 7.7	22 ± 8.4	*p* = .239
Latency to SWS (min)	112 ± 75	116 ± 14	172 ± 124	121 ± 122	59 ± 64	77 ± 70	*p* = .028
Duration of SWS (min)	48 ± 34	47 ± 31	43 ± 27	28 ± 25	44 ± 48	38 ± 43	*p* = .295
Duration of SWS (% of TST)	17 ± 13	18 ± 13	17 ± 14	10 ± 12	12 ± 12	10 ± 12	*p* = .994

The mean ± standard deviation (SD) of sleep parameters for both olanzapine and placebo treated group. TST = Total Sleep Time, SWS = Slow Wave Sleep, REM = Rapid Eye Movement. P value reported for the interaction (time by treatment) of a two-way repeated measures ANOVA.

**Figure 2:**
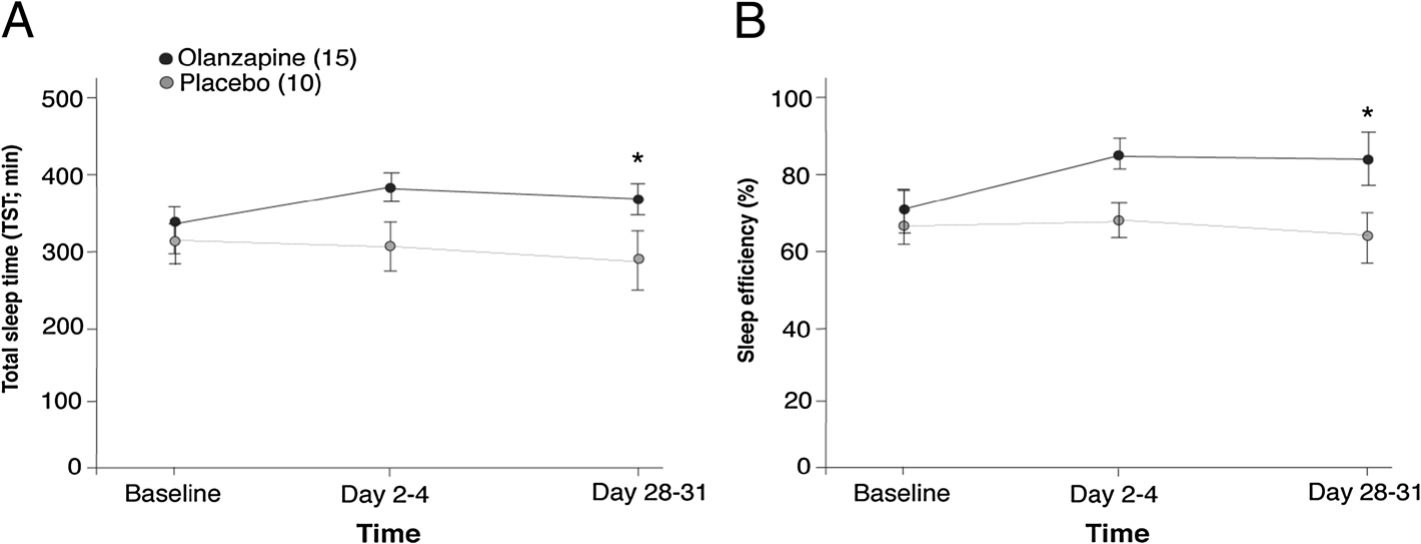
**Sleep continuity. A)** Total sleep time **B)** Sleep efficiency. Mean ± standard error of the mean for both olanzapine- and placebo-treated groups. Total sleep time is measured in minutes. Sleep efficiency: % = total sleep time(TST)/time in bed (TIB)× 100. *p < .05 between placebo and olanzapine groups on Day 28**–**31.

TST and Sleep efficiency are shown in Figure 2 for both olanzapine- and placebo-treated groups. Two two-way repeated-measures ANOVA showed an increase in TST (trend for main effect of treatment, F[1,23] = 3.69, p = 0.067) and sleep efficiency (main effect of treatment, F[1,23] = 5.10, p = 0.034) of the olanzapine-treated group compared to the placebo-treated group. Both TST and sleep efficiency demonstrated significant time x treatment interactions (TST: F[1,23] = 4.58; p = .043; sleep efficiency: F[1,23] = 5.27, p = 0.031). Post-hoc independent-samples t-tests were conducted to analyze the nature of these interactions and revealed no significant difference in TST or sleep efficiency at baseline (TST: t_23_ = -0.325, p = 0.798; Sleep efficiency: t_23_ = -0.460, p = 0.65) but by Day 28–31 olanzapine treatment significantly increased both (TST: t_23_ = -2.21; p = 0.037; Sleep efficiency: t_23_ = -3.30; p = 0.003).

As well as an increase in TST, olanzapine treated participants experienced significantly fewer awakenings (Table 2) and less overall time awake compared to treatment with placebo (2-way repeated measures ANOVA; main effect of treatment, number of awakenings, F[1,23] = 10.0, p = 0.004; awake time, F[1,23] = 4.8, p = 0.037). Post-hoc analysis of the significant interaction of time by treatment in awake time (F[1,23] = 4.93, p = 0.037) indicated that at baseline, awake times did not differ between placebo and olanzapine-treated participants (t_23_ = 0.531, p = 0.601) but by Day 28–31, olanzapine treatment was significantly decreasing amount of awake time participants experienced (t_23_ = 2.26, p = 0.003).

Two-way repeated measures ANOVA also analyzed the percentage of TST spent in R and duration of R (Table 2). There was neither a main effect of treatment or time on percentage of TST spent in R (F[1,23] = .796, p = 0.382; F[1,23] = .025, p = 0.875, respectively. There was a significant effect of olanzapine treatment on duration of R (F[1,23] = 4.78, p = 0.039) however there was neither a significant effect of time (F[1,23] = .003, p = 0.957) or a significant interaction of treatment by time (F[1,23] = .696, p = 0.413). Furthermore, Table 2 means indicate that the olanzapine group showed longer R than the placebo group even at baseline, suggesting that the difference in R sleep was not due to the addition of olanzapine.

### Measures

**Figure 3:**
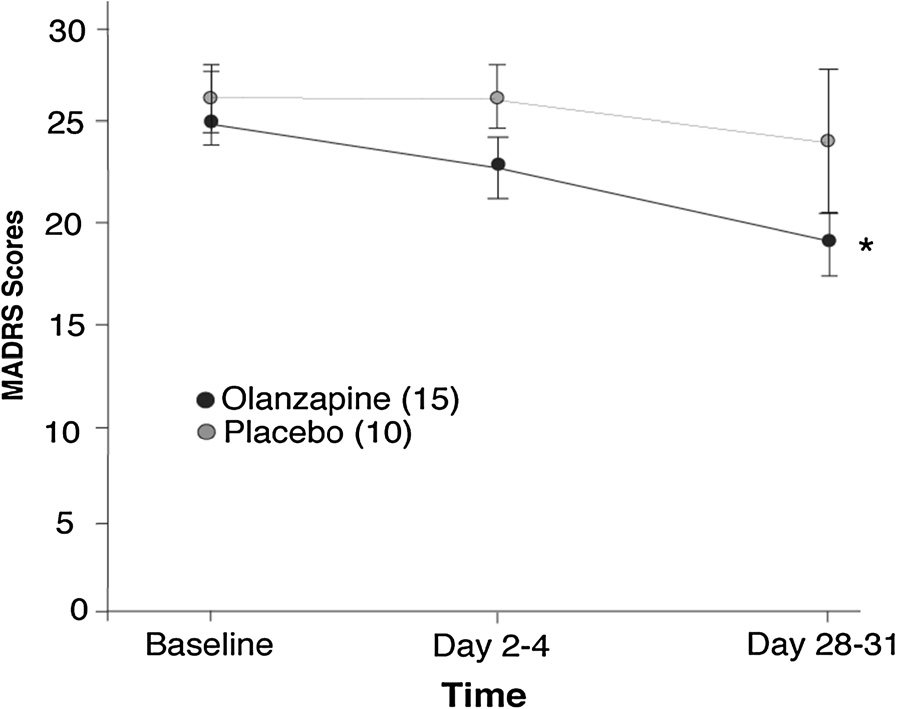
**Montgomery-Asberg Depression Rating Scale.** Total score with mean ± standard error of the mean for both olanzapine- and placebo-treated groups. *p < .001 for olanzapine groups across time.

Clinical measures are included as results of a secondary objective of the study. Figure 3 shows the mean MADRS total scores for both olanzapine- and placebo-treated participants. Two-way repeated ANOVA showed no significant differences between olanzapine and placebo treatment for MARDS or HDRS scores (F[1,23] = 1.09, p = .306; F[1,23] = 0.269, p = .609, respectively). However, both MARDS and HDRS scores significantly decreased with time (F[1,23] = 6.03, p = .022; F[1,23] = 20.0, p = <.001, respectively). One-way repeated measures ANOVA showed a significant decrease in total MADRS score from baseline to the end of the trial (day 28–31) for olanzapine-treated patients (main effect of time: F[1,14] = 6.03, p = .022), where as placebo treatment did not show a decrease (F[1,9] = 0.529, p = .486). HDRS scores also significantly decreased over time in the olanzapine-treated group (F[1,14] =19.6, p = .001), but the placebo-treated group did not (F[1,9] =4.67, p = .059).Table 3 shows clinician administered and self report rating scale scores for both the olanzapine- and placebo-treated groups.

**Table 3:** Clinical measures

**Clinical measures**	**Placebo**			**Olanzapine**			
	**Baseline**	**Day 2-4**	**Day 28-31**	**Baseline**	**Day 2-4**	**Day 28-31**	**ANOVA**
	**Mean ± SD**			**Mean ± SD**			
MADRS total	26 ± 6.3	26 ± 7.1	24 ± 3.2	25 ± 5.8	23 ± 6.2	19 ± 11	*p* = .175
HDRS total	21 ± 5.5	19 ± 5.9	18 ± 7.4	22 ± 5.0	18 ± 6.4	15 ± 8.4	*p* = .123
HARS total	16 ± 2.9	13 ± 4.7	13 ± 5.3	17 ± 5.8	15 ± 7.4	13 ± 6.7	*p* = .420
YMRS total	3.9 ± 3.4	3.7 ± 2.5	4.7 ± 4.2	3.7 ± 3.1	2.9 ± 3.0	3.5 ± 2.3	*p* = .223
PSQI total	13 ± 3.0	14 ± 3.6	12 ± 5.2	12 ± 3.5	11 ± 4.2	9.9 ± 4.7	*p* = .699
VAS total	31 ± 15	32 ± 19	43 ± 27	37 ± 27	45 ± 28	59 ± 30	*p* = .415
ESS total	7.4 ± 6.2	5.4 ± 4.2	5.8 ± 5.9	8.4 ± 5.2	8.5 ± 5.6	7.3 ± 5.2	*p* = .666

The mean ± standard deviation (SD) of selected clinical measures for both olanzapine and placebo treated group. MADRS = Montgomery Asberg Depression Rating Scale, HDRS = Hamilton Depression Rating Scale, HARS = Hamilton Anxiety Rating Scale, YMRS = Young Mania Rating Scale, VAS = Visual Analogue Scale for sleep quality, PSQI = Pittsburg Sleep Quality Index, ESS = Epworth Sleepiness Scale. P value reported for the interaction (time by treatment) of a two-way repeated measures ANOVA.

### Neurocognitive measures

Neurocognitive measures, evaluated as mean CANTAB testing scores, are shown in Table 4 and were assessed as a secondary objective of the study. The between errors and strategy score of the CANTAB SWM task of both the olanzapine- and placebo-treated groups did not significantly change between groups (F[1,22] = 0.3, p = 0.590; F[1,22] = 0.658, p = 0.426, respectively) or across time (F[1,22] = 1.4, p = 0.242; F[1,22] = 0.713, p = 0.407, respectively). The mean spatial span length recalled on the CANTAB SSP task was also not significantly different between treatment groups or across time (2-way repeated measures ANOVA, F[1,22] = 0.102, p = 0.753; F[1,22] = 0.036, p = 0.852, respectively). Finally, the mean reaction time and movement time from the CANTAB RTI task for both the olanzapine- and placebo-treated groups also showed no significant changes as a result of olanzapine treatment (2-way repeated measures ANOVA reaction time and movement: treamtment, F[1,22] = 0.089, p = .768; F[1,22] = 0.15, p = .702, respectively) or over time (2-way repeated measures ANOVA reaction time and movement: time, F[1,22] = 2.28, p = .145; F[1,22] = 2.57, p = .123, respectively).

**Table 4:** Cognitive measures

**Cognitive measures**	**Placebo**			**Olanzapine**			
	**Baseline**	**Day 2-4**	**Day 28-31**	**Baseline**	**Day 2-4**	**Day 28-31**	**ANOVA**
	**Mean ± SD**			**Mean ± SD**			
SWM							
Between errors	23 ± 26	22 ± 23	12 ± 15	34 ± 24	31 ± 19	21 ± 21	*p* = .778
Strategy score	29 ± 8.4	29 ± 7.6	28 ± 7.8	31 ± 8.0	30 ± 7.0	31 ± 6.9	*p* = .562
RTI							
Reaction time	410 ± 80.6	395 ± 43.3	405 ± 52	410 ± 110	380 ± 92.8	388 ± 111	*p* = .333
Movement time	579 ± 110	567 ± 104	539 ± 61	542 ± 132	563 ± 136	530 ± 107	*p* = .395
SSP	5.8 ± 1.4	5.7 ± 1.6	5.6 ± 1.6	5.4 ± 1.4	5.4 ± 1.2	5.7 ± 1.4	*p* = .296

The mean ± standard deviation (SD) of cognitive measures for both olanzapine and placebo treated group.SWM = Spatial Working Memory task, RTI = Reaction Time task, SSP = Spatial Span task. P value reported for the interaction (time by treatment) of a two-way repeated measures ANOVA.

## Discussion

Surprisingly, the addition of olanzapine to the current medication regimes of patients experiencing either unipolar or bipolar major depressive episodes only resulted in significant improvements in latency to SWS but not in other SWS measures, the primary outcome measure of this study. However, olanzapine augmentation did significantly improve the secondary outcome measure sleep continuity, that included sleep efficiency, number of awakenings, time spent awake and total sleep time. These improvements did not extend to significant changes other aspects of sleep architecture, like percentage of R sleep. Treatment with olanzapine also significantly improved illness severity without changes in measures of executive and psychomotor function.

To date, Sharpley *et al.*[13] have reported the only study to investigate the addition of olanzapine to SSRI treatment on overnight polysomnograph in patients with treatment resistant MDD. They showed significant improvements in sleep efficiency, total sleep time, percentage of time awake, total N percentage, subjective sleep quality, a decrease in R percent, and an increased R latency after a single dose of olanzapine. Our study revealed similar improvements only for sleep continuity measures and illness severity. We observed similar improvements in HDRS scores. The study by Sharpley *et al.*[13] included only patients with treatment resistant (to SSRI’s) MDD, and olanzapine was administered open-label with no control or comparison group. In comparison, in our randomized, double-blind and placebo controlled study, both patients with MDD and BD were included, with most patients that were recommended to receive augmentation to their antidepressant treatment being treatment-resistant to SSRI’s. Thus, conflicting findings may be due to the differences in the populations studied and methodology.

Antidepressants rarely improve sleep continuity; most improvements are seen with tricyclic antidepressants. Increased total sleep time and sleep efficiency, as well as decreased sleep latency and total wake time were observed here, similar to that seen with other augmentation agents such as risperidone [64], but different than quetiapine, which does not alter sleep continuity in depression [65]. The beneficial effects of olanzapine treatment on sleep continuity may be due to its diverse pharmacological profile. Primarily, olanzapine is an antagonist at 5-HT2A/C, 5-HT3 and 5-HT6 receptors [33]. A reduction in serotonin firing rates allows for the first sleep cycle to occur and serotonin suppresses R sleep by inhibition of R-promoting cholinergic neurons [6]. Olanzapine may also promote normalization of sleep through antagonism of overactive cholinergic neurons, as olanzapine has affinity for muscarinic M1-M5 receptors. Cholinergic neurons are responsible for the initial activation and ongoing generation of R sleep [6]. Olanzapine also has moderate affinity for the α-adrenergic1 receptor. The adrenergic system plays a role in the suppression of R and the promotion of sleep cycles [6]. Finally, olanzapine also has affinity for the histamine1 receptor. Histamine is thought to promote wakefulness and reductions in histamine allow for sleep to occur [29]. Therefore, olanzapine’s effects on neurotransmitter systems important for normal sleep continuity likely caused improvement in reported sleep continuity.

Few medications have been reliably shown to improve SWS in the treatment of either unipolar or bipolar depression. Only one randomized controlled trial (RCT) examining the effects of trazodone on sleep using polysomnography was identified. A study by Saletu-Zyhlarz *et al.*[66] examined insomnia patients with dysthymia and reported that trazodone administration significantly increased latency to stage 3, time and percentage in stage 3, sleep efficiency, and number of awakenings. A significant increase in the amount and percentage of R sleep was also seen. Bemmel *et al*. [67] conducted a single blind study of trazodone treatment on sleep architecture in patients with MDD. They reported no improvements to SWS and significant R suppression. In contrast, Mouret *et al.*[68] examined the effects of open-label trazodone on sleep in patients with MDD and reported significant improvements in SWS and no effect on R sleep. A study by Kluge *et al.*[69] examined the effects of duloxetine open-label on sleep in MDD, and reported a significant improvement in latency to SWS and time in SWS. Significant R suppression was reported. No RCTs on the effects of duloxetine were identified. Antidepressants reliably suppress R sleep; however, their effects on SWS are inconsistent in the treatment of major depressive episodes.

Almost all antidepressants have been shown to suppress R sleep by increasing the latency to R and decreasing the total duration of R sleep. R suppression was not seen in this study. Although expected, the lack of R suppression may be due to the concomitant medications of the participants. Only three participants were not taking any other medications, 80% of participants were taking at least one antidepressant, one participant was taking a benzodiazepine only, and one participant was taking lithium only. Over 50% of participants were taking more than one medication at enrolment into the study. The antidepressant medications and lithium may have suppressed R sleep and thus further suppression of R sleep with the addition of olanzapine might have been too minimal to detect. Although previous studies of augmentation to SSRIs have shown R suppression, the degree of polypharmacy was less in these studies, as each participant was only taking one antidepressant [70]. This may reflect the changes often seen with psychotropic treatment to sleep architecture [65,71]. Few medications have been reliably shown to suppress R sleep and also increase sleep continuity and SWS. Many medications, especially antidepressants, suppress R sleep and have either no effect or detrimental effects on sleep continuity or SWS. Also, several augmentation strategies, including olanzapine, have been shown to improve sleep continuity and SWS and have no effect on R sleep. Therefore, it is plausible that R suppression and other changes in sleep architecture are due to separate underlying mechanisms and may need to be treated separately.

The improvement of depressive symptoms observed in this study is representative of the effects of other atypical antipsychotic agents in the treatment of depression, as the olanzapine group had significantly greater improvement than placebo in the MADRS score. The power of the study, however, was not such as to determine efficacy. Clinical response was seen in 46% of olanzapine-treated patients while only 20% of placebo-treated patients did so. Twenty-six percent of olanzapine-treated participants reached remission and less than half of that (10%) in the placebo group did so. Previously reported response rates in a randomized double-blind study of olanzapine/fluoxetine combination for the treatment of depression are similar to what is observed here [36]. Significant improvement in depressive symptoms with olanzapine/fluoxetine combination versus placebo in the treatment of bipolar disorder has been reported as well [72].

The literature on cognitive function in depression is quite diverse and conflicting, as there seems to be no clear pattern of dysfunction in MDD [14,73,74]. The lack of changes seen here in working memory or psychomotor function was not unexpected. Impairments seen in neurocognitive tests in depression may be due to a lack of motivation to complete the task accurately and thus psychotropic treatment or sleep normalization would not have an effect on this beyond improvement of depressive symptoms. As only 26% of our participants reached remission, many participants had residual depressive symptoms and may not have had an improvement in motivation. The lack of a normal control group precludes us from reporting if the level of baseline functioning is within the normal range or is impaired and did not improve with treatment.

There are a number of limitations in this study. First, participants were maintained on a large variety of concomitant medications. This likely contributed to the highly variable effect of olanzapine on SWS. Secondly, there were a large number of secondary outcome measures and a relatively small sample size. This is most relevant to the lack of change seen in SWS, R sleep and on the neurocognitive measures. A larger sample size may also lend itself to a more precise differentiation between placebo- and olanzapine-treated participants on improvements in subjective sleep quality. Furthermore, the duration of this study only permitted evaluation of the short-term treatment of depression; it is well understood that to observe full improvement with many different psychotropic medications 6–8 weeks or even longer is needed. Finally, the results of this study are generalizable to a select number of patient populations, as there were some comorbid exclusions and a variety of concomitant medications. For instance, the results of this study do not allow us to understand what effect olanzapine may have on sleep architecture in patients with comorbid substance abuse. Further studies should evaluate the influence of different dosages and long-term studies are needed to see if improvements continue into treatment maintenance.

## Conclusions

Olanzapine augmentation in the treatment of both unipolar and bipolar depression resulted in significant improvements in sleep continuity, total sleep time and latency to sleep onset as well as significantly improved depressed mood. However, significant changes were only moderately seen in SWS and not at all in R sleep. Olanzapine has been shown to increase SWS in other psychiatric disorders, e.g., schizophrenia [75]. Factors as simple as dosage of olanzapine may moderate affects on SWS across patient populations. Furthermore, even within patient populations much of the literature shows conflicting results [66-71] of olanzapine on sleep architecture. Changes in cognition, both working memory and psychomotor function, were not observed. However, olanzapine may be one of few medications that improve sleep continuity, thus directly targeting symptoms of depression. Olanzapine may produce this effect through its high affinity for not only serotonin receptors but also muscarinic, adrenergic and histaminergic receptors. The heterogeneity of this study population allows for generalizability to clinical populations, and as such olanzapine may be an effective tool in the treatment of sleep dysfunction in depression.

## Abbreviations

MADRS: Montgomery-asberg depression rating scale; CANTAB: Cambridge neuropsychological test automated battery; SWM: Spatial working memory; SSP: Spatial span; RTI: Reaction time; MDD: Major depressive disorder; BD: Bipolar disorder; R: Rapid eye movement; N: Non-REM; SWS: Slow wave sleep; TST: Total sleep time; EEG: Electroencephalogram; NA: Norepinephrine; 5-HT: Serotonin; MINI: Mini International neuropsychiatric inventory; MDE: Major depressive episode; HDRS-17: Hamilton depression rating scale-17; YMRS: Young mania rating scale; HARS: Hamilton anxiety rating scale; PSQI: Pittsburgh sleep quality index; VAS: Visual analogue scale; ESS: Epworth sleepiness scale; CGI-S: Clinical global impression-severity; CGI-I: Clinical global impression-improvement; PSG: Polysomnograph; EMG: Electromyogram; RDI: Respiratory disturbance index; SD: Standard deviation; SSRI: Selective serotonin reuptake inhibitor; RCT: Randomized controlled trial.

## Competing interests

The study was funded by a research fellowship from Eli Lilly Canada as awarded to L. Lazowski under the supervision of Dr. R. Milev. BT, ERH, RJ RT declare that they have no competing interests.

## Authors’ contributions

LL contributed substantially to the conception and design, acquisition of data, drafting the article and gave final approval of the version to be published. BT contributed substantially to the analysis and interpretation of the data, drafting the article and gave final approval of the version to be published. ERH contributed substantially to the analysis and interpretation of data, revised it critically for important intellectual content and gave final approval of the version to be published. RJ contributed substantially to the conception and design, acquisition of data, drafting the article and gave final approval of the version to be published. RT contributed substantially to the conception and design, acquisition of data, drafting the article and gave final approval of the version to be published. RM contributed substantially to the conception and design, revised it critically for important intellectual content and gave final approval of the version to be published.

## Pre-publication history

The pre-publication history for this paper can be accessed here:

http://www.biomedcentral.com/1471-244X/14/202/prepub
